# Chlorhexidine Improves Hygiene Reducing Oral Polymorphonuclear Leukocytes with Antimicrobial Effects at Distinct Microenvironments amongst Subjects Stratified by Health Status

**DOI:** 10.3390/antibiotics11050603

**Published:** 2022-04-29

**Authors:** Prem K. Sreenivasan, Violet. I. Haraszthy

**Affiliations:** 1 HITLAB, 3960 Broadway, New York, NY 10032, USA; 2 Department of Oral Biology, Rutgers School of Dental Medicine, Newark, NJ 07103, USA; 3 Department of Restorative Dentistry, University at Buffalo, Buffalo, NY 14214, USA; vh1@buffalo.edu

**Keywords:** chlorhexidine, dental plaque, gingivitis, oral hygiene, polymorphonuclear leukocytes [PMN]

## Abstract

Oral conditions such as gingivitis and oral malodor are commonly reported globally. **Objective:** This investigation clinically stratified subjects to healthy, malodor and gingivitis groups and enumerated oral polymorphonuclear leukocytes (PMN) as a measure of inflammation prior to and after rinsing with a chlorhexidine (CHX) mouthwash. The study also assessed clinical outcomes (dental plaque and gingival bleeding indices), malodor (halimeter scores, organoleptic and tongue coat index and microbiological parameters (anaerobic and malodor organisms of dental plaque, tongue surface and saliva) for a comprehensive assessment of the oral inflammatory burden. **Methods:** Consenting adults were stratified into control (*n* = 17), gingivitis (*n* = 19) and halitosis (*n* = 17) groups based on clinical criteria. At baseline, oral samples were examined for PMN in addition to microbiological analysis of dental plaque, saliva and tongue scrapings for anaerobic and malodor bacteria. Subjects were issued a commercially available fluoride toothpaste and a chlorhexidine mouthwash for two-week use prior to post-treatment assessments identical to baseline. **Results:** At baseline, PMN were lowest in the control that increased amongst the halitosis subjects, with the gingivitis group registering the highest levels (*p* < 0.05) with these outcomes corresponding with clinical parameters (*p* < 0.05). CHX use improved outcomes with a 56–61% reduction in PMN with significant differences between groups (*p* < 0.05). Dental plaque and bleeding indices were lower by 57–78% with oral malodor, demonstrating reductions of 33–59% (*p* < 0.05). Significant reductions in anaerobic and malodor organisms ranging from 78–96% and 76–94%, respectively, were noted after CHX use (*p* < 0.05). **Conclusions:** At study enrollment, PMN scores were lowest in healthy subjects, with increasing numbers amongst halitosis followed by gingivitis. Amongst all subject groups, CHX use significantly reduced oral PMN and corroborated with corresponding decreases in clinical, malodor and bacterial outcomes. Together, these results demonstrate the significant reductions in the oral inflammatory burden following CHX use.

## 1. Introduction

A variety of common oral health problems reported globally include inflammatory conditions such as gingivitis [1]. In the absence of effective treatment, gingivitis may progress to periodontal disease with distinct clinical observations, such as the loss of bone and tissue supporting the teeth [2]. In addition to these conditions, oral malodor is also reported world-wide and associated with oral aesthetics [1,2]. Oral malodor is associated with important multifactorial influences and has been linked to the psychological and social lives of those afflicted [3,4,5,6,7,8].

Oral inflammation, representing a clinical observation in gingivitis and malodor, is attributed to several factors, including the large densities of organisms found within the distinct regions of the human mouth [1,3,7,8]. Oral organisms are commonly found as sessile biofilms on the surfaces of the exposed teeth as supragingival plaque, on the surfaces of the tongue, cheeks, gums and palate [1,2,3,8]. In addition, oral bacteria are found as planktonic populations in the saliva that transmit the organisms within the distinct regions of the mouth [1,3,4,5,6,7,8]. Notable features of oral inflammation include the influx of polymorphonuclear leukocytes [PMN] that transit from the vasculature through the epithelium and oral mucosa [9,10,11]. Following transit, PMN are found in mucosal secretions [12] and in the saliva [13]. Important features of PMN include their substantial density as effector cells and they are commonly referred to as “first-responders” [14]. Neutrophils are identified in estimating inflammation, microbial burden and reported in samples such as nasal [15,16,17], respiratory [18], eye [19] and other regions [20] including inflammation due to occupational exposure [16]. With reference to samples relevant to dentistry, neutrophils are reported from pediatric [21] and adult populations [11].

Whereas current priorities in dentistry emphasize education, outreach and routine dental care to improve oral health and hygiene, inadequate hygiene remains commonplace [1,2,3,4,5,6,7,8,22]. Formulations that augment hygiene by the incorporation of antimicrobial agents in mouthrinses are additional adjuncts to maintain oral hygiene [23]. Amongst the various antimicrobial agents, the features of chlorhexidine [CHX], a cationic bisbiguanide with broad-spectrum effects is well established [1,7,23,24]. Based on the available evidence, CHX is considered the gold-standard with applications in medicine [24] and dentistry [1,7,23].

This investigation was designed with a few goals. Primary amongst them was an assessment of oral PMN as a measure of inflammation amongst subjects clinically stratified as healthy, gingivitis or malodor. The effects of rinsing with a CHX mouthrinse on oral PMN were elucidated amongst these subjects. Additionally, this study included clinical evaluations for dental plaque, bleeding index and periodontal pocket depths using established indices that were conducted prior to and after CHX rinsing. Oral malodor assessments represented by organoleptic evaluations, halimeter assessments and tongue coat index were determined prior to and after use of CHX. The effects of CHX on the oral microbial burden of dental plaque, tongue surface and saliva enumerating anaerobic and malodor organisms in these samples prior to and after CHX use represented the microbiological objective. Together, the study was designed to provide multi-parameter assessments amongst subjects stratified clinically.

## 2. Results

### 2.1. Clinical and Demographic Data

Fifty-three subjects (19 males and 34 females; average age 45.72 years) were enrolled and these subjects completed the study without adverse events. A summary of the age and gender of the study population is presented in Table 1. The three groups of subjects did not differ significantly with respect to age or gender (*p* > 0.05) evaluated by ANOVA and chi-square, respectively. Throughout the study, there were no adverse effects on the oral soft or hard tissues observed by the examiner, or reported by the subjects.

### 2.2. Oral Neutrophil Evaluations

Oral neutrophil scores over the study period are shown in Table 2. Treatment groups demonstrated differences in neutrophil scores at baseline, with the gingivitis group registering the highest mean scores (*p* < 0.05). All clinical groups demonstrated significant reductions from their corresponding baselines, with reductions ranging from 56–61% for oral neutrophils 12 h after CHX use (*p* < 0.05). Additionally, analyses indicate significant differences at the post-treatment evaluation between treatment groups (*p* < 0.05).

### 2.3. Clinical Evaluations

A summary of clinical parameters representing whole mouth dental plaque and bleeding index assessments from treatment groups in conjunction with periodontal pocket depths is summarized in Table 3. At baseline, dental plaque results did not demonstrate significant differences between treatment groups (*p* > 0.05). Bleeding index results from the control group were significantly different from the halitosis group (*p* < 0.05), with the gingivitis group demonstrating no significant differences from either the control or the halitosis groups (*p* > 0.05). Periodontal pocket depths were significantly higher amongst the gingivitis and halitosis groups in comparison to the control at baseline (*p* < 0.05). At the post-treatment evaluation, conducted 12 h after rinsing with CHX, all groups demonstrated significant reductions for dental plaque, bleeding index and periodontal pocket depths from their corresponding baseline (*p* < 0.05). Bleeding index outcomes demonstrated significant differences between the control and halitosis groups at the post-treatment evaluations (*p* < 0.05). Periodontal pocket depths registered no significant differences between the treatment groups (*p* > 0.05). For the treatment groups, percentage reductions in plaque index outcomes ranged between 61–74% at the post-treatment evaluation with bleeding index results registering between 57–78% from their corresponding baselines (*p* < 0.05). Periodontal pocket probing depths were reduced between 8–16% at the post-treatment evaluation for the treatment groups from their corresponding baselines (*p* < 0.05).

### 2.4. Malodor Assessments

Malodor parameters recorded over the study period are summarized in Table 4. At baseline, the organolepic and tongue coat index demonstrated significant differences between clinical groups (*p* < 0.05) with the halitosis group registering significantly higher halimeter scores at baseline than all other groups (*p* < 0.05). At the post-treatment evaluation, treatment groups registered reductions in malodor outcomes from their corresponding baselines (*p* < 0.05) with the exception of halimeter scores for the control group (*p* > 0.05). Post-treatment scores for the halitosis group were higher than the control at the post-treatment evaluation with percentage differences for these outcomes ranging from 33–59%.

### 2.5. Microbiological Assessments

Microbiological analyses for anaerobic and malodor organisms in dental plaque, saliva and tongue surface samples are presented in Table 5, Table 6 and Table 7. With the exception of anaerobic plaque organisms at the baseline visit that demonstrated differences between the clinical groups (*p* < 0.05), no differences were registered in all other outcomes at baseline (*p* > 0.05). Irrespective of clinical group, the use of CHX demonstrated significant reductions in all microbial parameters from their corresponding baseline (*p* < 0.05). Plaque anaerobic bacteria demonstrated reductions of 91–96% and concomitant reductions between 77–94% for malodor organisms after CHX treatment (*p* < 0.05). In salivary samples, anaerobic organisms registered reductions between 78–83% and malodor bacteria between 76–81% from their respective baselines (*p* < 0.05). Tongue surface anaerobic bacteria and malodor organisms registered between 77–85% and 66–79%, respectively, after CHX treatment representing statistically significant outcomes (*p* < 0.05).

## 3. Discussion

Common oral conditions are identified globally with gingivitis and malodor described broadly [1,2,4]. While inflammatory features are important observations in both gingivitis and halitosis, a clinical assessment of these populations in conjunction with neutrophils representing critical effector cells of the immune response remains unexplored. Neutrophils regarded as the first-responders [14] are identified in mucosal interfaces including the eye [19], nose [15,16,17], respiratory [17] and other regions [12]. The substantial density of neutrophils and their ability to exit the vasculature represent features associated with their response to the inflammatory burden [14].

A comprehensive evaluation of PMN numbers amongst subjects stratified by clinical criteria into control, gingivitis and malodor groups in conjunction with clinical parameters for dental plaque and bleeding index, along with periodontal pocket probing depths, comprised important aspects of this investigation. These clinical outcomes find application to evaluate the effects of interventional strategies. While these clinical outcomes present an extended history, studies identify drawbacks including their subjectivity, patient discomfort and semi-quantitative outcomes [25]. Clinical assessments for malodor included organoleptic measures, halimeter scores and a tongue coat index representing accepted indices were evaluated. Microbiological analyses of oral samples collected from dental plaque, saliva and tongue surface that were examined for anaerobic organisms and malodor bacteria augmented clinical outcomes. Together, these efforts were designed to include objective measures of treatment effects and an assessment of the oral inflammatory burden. Test conditions for the study were standardized with a two-week washout phase test prior to baseline assessments to reduce the influences of previously utilized oral hygiene formulations. From the stand-point of study design, the shorter duration of the interventional portion of the study with CHX representing a well-recognized oral therapeutic [1,23,24], along with periodic follow-ups with study subjects, facilitated monitoring of adverse events while providing subject reminders on study procedures.

At baseline, treatment groups registered significant differences in PMN scores representing new findings of clinical relevance amongst stratified subjects. PMN scores were significantly different between treatment groups, with the gingivitis group registering an average score of 5.85. The halitosis group with a score of 5.62 was significantly lower than gingivitis subjects with the lowest PMN outcomes observed amongst the healthy controls (*p* < 0.05). While clinical measures at baseline between the treatment groups broadly followed the PMN outcomes, it is striking to note statistically significant differences between treatment groups based on neutrophil assessments. Neutrophil results within treatment groups aligned broadly with bleeding index scores, with the highest average baseline scores recorded amongst subjects assigned to the gingivitis and halitosis groups. For instance, average baseline dental plaque index scores were higher amongst the gingivitis and halitosis groups in comparison to the control (*p* < 0.05). Average pocket depth results were also higher amongst the gingivitis and halitosis clinical groups in comparison to the control (*p* < 0.05).

CHX treatment resulted in significant reductions for PMN amongst all clinical groups (*p* < 0.05). Differences in PMN results at baseline between treatment groups were maintained at the post-treatment observations. With the lowest PMN scores of 4.75 amongst the control group, higher PMN scores were noted in the gingivitis and halitosis groups (*p* < 0.05). These outcomes are noteworthy in representing prominent treatment differences between the distinct treatment groups. At the post-treatment evaluations, PMN results demonstrated percentage reductions between 56–61% from corresponding baselines. While post-treatment outcomes from PMN demonstrated similarities with bleeding index, dental plaque index and periodontal pocket depths, the differences between treatment groups for bleeding index evaluations are noteworthy. Furthermore, while bleeding index scores for the control were the lowest at the post-treatment evaluation, they were significantly different from the halitosis group (*p* < 0.05) but not significantly different from the gingivitis group (*p* > 0.05). On the other hand, PMN outcomes represented significant differences between treatment groups. Therefore, the observed differences between PMN and bleeding index results at the post-treatment evaluations is indicative of greater treatment differentiations from PMN evaluations. An ability to identify such differences in a moderately sized treatment population provides flexibilities in designing studies, including those evaluating interventional strategies. Laboratory methods for PMN enumeration, sample analyses and handling afford many additional flexibilities. Advances in cytometry, automation and PMN based point-of-care diagnostics will facilitate rapid reporting and chair-side analyses to support patient education and oral health management.

Malodor outcomes representing organoleptic, halimeter and tongue coat index at the post-treatment evaluations were consistently lower for each clinical group from their corresponding baselines. While the control group consistently registered the lowest post-treatment scores, there were few additional differentiators. The control group, while significantly lower than the halitosis cohort for the tongue coat index, were not significantly different for the organoleptic or halimeter outcomes. Additionally, there were no significant differences between the gingivitis or halitosis groups for malodor parameters (*p* > 0.05).

Microbiological assessments at the post-treatment evaluation showed marked reductions for both anaerobic and malodor organisms in oral reservoirs, i.e., dental plaque, saliva and tongue surface in comparison to baseline (*p* < 0.05). These sites were selected based on the identified influences of microorganisms in these regions on the selected clinical groups, i.e., gingivitis and malodor, and evaluated using previously described methods [26,27,28]. Outcomes of relevance from the microbiological analysis include the lower baseline numbers of organisms in the control group for both anaerobic and malodor organisms in each oral environment in comparison to both the gingivitis and halitosis populations. The control group also maintained the lower numbers of bacteria at the post-treatment examination. Rinsing with CHX resulted in broad reductions for all microbial parameters. Anaerobic organisms demonstrated reductions between 77–96%, with reductions of 66–94% for malodor organisms in evaluated samples. Although not statistically significant, the halitosis group consistently demonstrated the highest numerical reduction for both anaerobic and malodor organisms, irrespective of evaluated sample. CHX finds wide application in dental practice and amongst consumer products for oral hygiene, representing the gold-standard with many investigations reporting its microbiological efficacy in clinical studies [1,23,24]. Reports of the lack of efficacy within the depths of the microbial biofilm and relative reductions in microbial susceptibility amongst isolated strains of organisms are available in controlled laboratory evaluations, leading to suggestions for additional research to monitor and safeguard the widely accepted clinical benefits of CHX [29].

Distinctive aspects of this investigation included determination of treatment effects 12 h after oral hygiene with a commercially available 0.12% chlorhexidine amongst clinically stratified subjects. A control group of subjects with no clinically identifiable characteristics comprised an important treatment group included in this investigation. While the study did not include a control treatment, it is important to highlight the available clinical studies documenting the clinical effects of CHX [23]. Additionally, studies have reported the effects of CHX on PMN and clinical parameters over a two-week period amongst gingivitis subjects in comparison to a control treatment [30] and on microbiological parameters [31].

## 4. Materials and Methods

### 4.1. Ethics

This single site, parallel design study was conducted after the protocol was approved by the ethics board of the University at Buffalo in compliance with all regulations and in accordance with the Declaration of Helsinki. Subject enrollment, clinical evaluations and sampling were conducted at the dental clinics of the University.

### 4.2. Patients and Study Design

Volunteers from the local area representing members of either gender between the age of 18–70 years who expressed an interest in study participation were provided relevant study information. Those voluntarily providing informed consent were scheduled for a screening visit at the dental clinic. The screening visit conducted by a dental professional included an interview on medical history and an oral examination of the soft and hard tissues. Subjects were evaluated for dental plaque, bleeding index, pocket depth and oral malodor using organoleptic methods, a halimeter assessment, and tongue coat index. Study enrollment was restricted to those presenting with 20 natural teeth and who were not undergoing medical or dental treatments. Subjects with systemic diseases, ongoing or impending pregnancy, participation in any clinical study in the preceding three months or under the care of a medical or dental profession requiring prescription medications were excluded. Also excluded were subjects with restorations, dental implants, orthodontic bands and dentures. Those presenting with carious lesions, soft tissue pathologies, ulcers and other symptoms requiring immediate care were referred to the dental clinics.

Based on an oral examination that evaluated the dental plaque index, bleeding index and malodor scores, subjects were placed into three groups, i.e., control [slight gingivitis with dental plaque and bleeding index scores less than 1.0 along with organoleptic scores that were less than 3], gingivitis [moderate gingivitis with dental plaque and bleeding index scores greater than 1.0 and organoleptic malodor scores less than 3.0] and halitosis [subjects who registered dental plaque and bleeding index scores greater than 1.0 in addition to organoleptic malodor scores greater than 3.0].

After study enrollment, subjects were provided a commercially available fluoride toothpaste and soft-bristled toothbrush for oral hygiene during the two-week washout phase. Enrolled subjects were instructed to discontinue the use of all other oral hygiene aids for the study period, provided a study schedule and instructed to arrive at the dental clinic at the conclusion of their washout phase for their baseline visit. Subjects refrained from oral hygiene for 12 h and from eating and drinking for four hours prior to their baseline visit with all evaluations conducted in the morning. During the baseline visit, subjects were evaluated for all parameters evaluated during the enrollment visit. In addition, an oral rinse sample was collected to evaluate neutrophils, a marker of oral inflammation.

### 4.3. Treatment Assignments

At the conclusion of the baseline evaluations, subjects were provided a commercially-available fluoride toothpaste and a 0.12% chlorhexidine [CHX] mouthwash for twice daily oral hygiene over the next two-weeks. At-home use instructions for the study subjects consisted of using provided articles and to refrain from any other oral hygiene procedures throughout the duration of the study. After brushing their teeth, subjects were instructed to rinse with the provided mouthwash for 30 s with 15 mL of mouthrinse. There were no other restrictions regarding diet or smoking habits during the course of the study.

### 4.4. Evaluations

Oral soft and hard tissue assessments, as well as clinical evaluations for dental plaque [32], bleeding index [33], pocket depth [25,34], malodor examinations by organoleptic assessments [2,3,4,35], halimeter [2,3,4,5,6,7,26,28,35], tongue coat index [36] and evaluations of neutrophil and bacteria of dental plaque, saliva and tongue surface were conducted at baseline and after two-week use of assigned treatment. Subjects were scheduled to return to the clinical facility for the post-treatment visit, having refrained from all oral hygiene procedures for twelve hours and from eating and drinking for at least four hours prior to their scheduled visit. All examinations were performed by the same dental examiner, using the same procedures as employed at baseline. Subjects were also interviewed with respect to adverse events and the use of concomitant medications.

### 4.5. Sample Collections

Collection of oral rinse samples for neutrophil evaluations: Subjects were provided 10 mL of sterile buffer in tubes marked with their initials. Subjects rinsed with this rinse for 15 s and expectorated contents into the tube. This sample was used for neutrophil evaluations.

Collection of dental plaque, saliva and tongue surface samples for microbiological evaluations:

Supragingival plaque: These samples were collected randomly from the buccal surfaces of the upper right or left quadrant (teeth # 2-8 or teeth # 9-15) using a sterile Columbia 13/14 scaler. Samples were pooled, and placed in a sterile tube [marked with subject identification details] containing 1 mL of suitable isotonic buffer such as Ringer’s solution or phosphate buffered saline (PBS).

Tongue scrapings: These samples were collected using the edge of a wooden tongue blade. A site was randomly chosen for each assessment. Each sample collection entailed five scrapes per site and samples transferred to a tube for microbiological evaluations. Samples were placed in a sterile tube [marked with subject identification details] containing 3 mL of suitable isotonic buffer such as Ringer’s solution or phosphate buffered saline (PBS).

Saliva sample: A sample of unstimulated saliva was collected from subjects in sterile tubes marked with subject identification details.

### 4.6. Laboratory Procedures with Collected Samples

Evaluation of neutrophils: All collected rinse samples were evaluated for neutrophils. Samples were gently mixed and diluted in 10-fold dilutions in PBS for evaluations. Microscopic evaluations were conducted on dilutions and results expressed as counts per milliliter (counts/mL). Results were log_10_ transformed for analysis as described previously [30,37,38].

Evaluation of oral bacteria in samples of dental plaque, saliva and tongue scrapings: All samples were briefly sonicated prior to serial 10-fold dilutions in saline. Dilutions were plated in duplicate on agar enriched with 5% sheep blood [26,27,31] and on agar to enumerate malodor organisms [26,27,28,31]. Plates were incubated under anaerobic conditions at 37 °C. The colony forming units (CFU) were calculated for analysis.

### 4.7. Statistical Analysis

Treatment groups were compared with respect to gender were performed using a chi-square analysis and by an analysis of variance (ANOVA) for age. Statistical analyses were conducted separately for each evaluated parameter, i.e., dental plaque index, bleeding index, pocket depth, malodor scores from organoleptic, halimeter evaluations and tongue coat index. Further, statistical evaluations were conducted separately for neutrophil and microbiological outcomes, i.e., anaerobic and malodor organisms of dental plaque, saliva and tongue surface with each of these results log_10_ transformed for analysis. Comparisons of the treatment groups with respect to baseline dental plaque index, bleeding index and malodor scores from organoleptic, halimeter evaluations and tongue coat index, pocket depths, neutrophils and microbiological outcomes for anaerobic and malodor bacteria of dental plaque, saliva and tongue surface were performed using an analysis of variance (ANOVA).

Within-treatment comparisons of the baseline versus follow-up dental plaque index, bleeding index, pocket depth, malodor scores from organoleptic, halimeter evaluations, tongue coat index, along with neutrophil and microbiological parameters for each group of organisms in distinct oral samples were performed using paired *t*-tests. All statistical tests of hypotheses were two sided, and employed a level of significance of α = 0.05.

## 5. Conclusions

The present study provides simultaneous assessments of clinical, immunological and microbiological outcomes for a comprehensive and objective assessment of treatment effects on the inflammatory burden. Objective measures of treatment outcomes can augment evaluations of therapeutic strategies to prevent or control common oral conditions. The chronic nature of common oral diseases and relationships to systemic outcomes [1,5,8] provide rationale for the current investigation that enrolled distinct groups of subjects and supports the development of future oral health monitoring initiatives. Biomarkers based on PMN are cleared by the FDA for clinical use [39] to evaluate mucosal healing and the clinical course of ulcerative colitis. Similarly, nasal cytology for PMN is highlighted for its clinical correlation with inflammation, with advantages that include cost-effectiveness and non-invasive point of care applications [15]. In this regard, these efforts identify with the priorities in dentistry.

## Figures and Tables

**Table 1:** Summary of Age and Gender for subjects completing the clinical study.

Subject Groups Based on Clinical Status	Number of Subjects ^1^	Number of Males	Number of Females	Mean AGE ^2^	Age (SEM)	Age (SD)
Control	17	4	13	40.59	4.20	17.32
Gingivitis	19	6	13	44.42	3.17	13.83
Halitosis	17	9	8	52.21	3.51	14.49

^1^ No statistically significant differences between subject groups for gender by chi-square analysis (*p* = 0.182). ^2^ No statistically significant differences between subject groups for age by ANOVA (*p* = 0.083).

**Table 2:** Summary of neutrophil scores (Log Counts/mL) for subjects completing the clinical study.

Subject Groups Based on Clinical Status	Baseline Visit ^§^	2-Week Post-treatment Visit ^ǂ^	Percent Reductions from Corresponding Baseline ^¶^
Control	5.17 ± 0.39	4.75 ± 0.32	61.58
Gingivitis	5.85 ± 0.41	5.44 ± 0.52	61.29
Halitosis	5.62 ± 0.50	5.26 ± 0.45	56.25

**^§^** Clinical groups demonstrated statistically significant differences for neutrophil scores at baseline (*p* < 0.05). **^ǂ^** Clinical groups demonstrated statistically significant differences for neutrophil scores at the two-week post treatment visit (*p* < 0.05). **^¶^** Significantly different from corresponding baseline by paired *t*-test for evaluated parameter (*p* < 0.05).

**Table 3:** Summary of dental plaque, bleeding index and pocket probing depths from subjects who completed the entire study.

Clinical Assessment	Treatment Groups Based on Clinical Status	Baseline Visit	2-Week Post-Treatment Visit	Percent Reduction from Corresponding Baseline
Dental Plaque	Control	0.88 ± 0.73	0.22 ± 0.31 ^¶^	74.10
	Gingivitis	1.51 ± 0.84	0.58 ± 0.62 ^¶^	61.25
	Halitosis	1.56 ± 0.75	0.50 ± 0.25 ^¶^	67.40
Bleeding Index	Control	0.76 ± 0.69 ^a^	0.16 ± 0.18 ^a,¶^	78.75
	Gingivitis	1.44 ± 0.86 ^a,b^	0.61 ± 0.57 ^a,b,¶^	57.36
	Halitosis	1.42 ± 0.79 ^b^	0.38 ± 0.26 ^b,¶^	73.14
Periodontal Pocket depth scores (mm)	Control	2.62 ± 0.38 ^a^	2.37 ± 0.28 ^a,¶^	8.81
	Gingivitis	3.05 ± 0.55 ^b^	2.58 ± 0.46 ^a,¶^	15.58
	Halitosis	3.09 ± 0.56 ^b^	2.55 ± 0.34 ^a,¶^	16.96

^a,b^ Subject groups that share alphabets demonstrate no statistically significant differences between subject groups (*p* > 0.05) in contrast to groups that do not share superscripts at each evaluation by ANOVA (*p* < 0.05). ^¶^ Significantly different from corresponding baseline by paired *t*-test for evaluated parameter (*p* < 0.05).

**Table 4:** Summary of malodor parameters from subjects who completed the entire study.

Clinical Assessment	Treatment Groups Based on Clinical Status	Baseline Visit	2-Week Post-Treatment Visit	Percent Reduction from Corresponding Baseline
Organoleptic	Control	1.46 ± 1.12 ^a^	0.60 ± 0.82 ^¶,d,e^	59.10
	Gingivitis	2.47 ± 1.25 ^b^	1.19 ± 1.25 ^¶,d,e,f^	51.94
	Halitosis	3.68 ± 0.88 ^c^	1.63 ± 1.01 ^¶,e,f^	55.70
Halimeter	Control	79.53 ± 30.85	52.87 ± 31.08	33.52
	Gingivitis	111.62 ± 39.89	51.00 ± 36.63 ^¶^	54.31
	Halitosis	253.5 ± 73.1 ^a^	107.7 ± 55.1 ^¶,§^	57.51
Tongue Coat Index	Control	1.47 ± 1.12 ^a^	0.70 ± 1.04 ^¶,g^	59.99
	Gingivitis	2.52 ± 0.77 ^b^	1.68 ± 1.15 ^¶,g,h^	38.18
	Halitosis	3.05 ± 0.74 ^c^	2.05 ± 1.34 ^¶,g,h^	43.47

^a–h^ Subject groups that share alphabets demonstrate no statistically significant differences between subject groups (*p* > 0.05) in contrast to groups that do not share superscripts at each evaluation by ANOVA (*p* < 0.05). ^§^ Significant differences between halitosis and all other groups (*p* < 0.05). ^¶^ Significantly different from corresponding baseline by paired *t*-test for evaluated parameter (*p* < 0.05).

**Table 5:** Summary of dental plaque bacteria (Log CFU/mL) for subjects who completed the clinical study.

Bacteria Evaluated	Treatment Groups Based on Clinical Status	Baseline Visit	2-Week Post-Treatment Visit ^¶^	Percent Reduction from Corresponding Baseline
Anaerobic plaque organisms	Control	6.91 ± 0.56 ^a^	5.84 ± 0.74 ^¶^	91.39
	Gingivitis	7.37 ± 0.58 ^a,b^	6.32 ± 0.92 ^¶^	91.11
	Halitosis	7.6 ± 0.60 ^b^	6.13 ± 0.68 ^¶^	96.61
Malodor plaque organisms	Control	6.32 ± 0.52 ^ǂ^	5.67 ± 0.87 ^¶^	77.51
	Gingivitis	6.52 ± 0.37 ^ǂ^	5.88 ± 0.65 ^¶^	77.08
	Halitosis	6.69 ± 0.44 ^ǂ^	5.45 ± 0.81 ^¶^	94.23

^a,b^ Subject groups that share alphabets demonstrate no statistically significant differences between subject groups (*p* > 0.05) in contrast to groups that do not share superscripts by ANOVA (*p* < 0.05). ^ǂ^ No significant differences between treatment groups for evaluated organism at the baseline visit (*p* > 0.05). ^¶^ Significantly different from corresponding baseline by paired *t*-test for evaluated organism (*p* < 0.05).

**Table 6:** Summary of salivary bacteria (Log CFU/mL) for subjects who completed the clinical study.

Bacteria Evaluated	Treatment Groups Based on Clinical Status	Baseline Visit ^ǂ^	2-Week Post-Treatment Visit ^¶^	Percent Reduction from Corresponding Baseline
Anaerobic salivary organisms	Control	7.61 ± 0.59	6.91 ± 0.75 ^¶^	80.41
	Gingivitis	7.71 ± 0.51	7.04 ± 0.55 ^¶^	78.47
	Halitosis	7.93 ± 0.41	7.16 ± 0.58 ^¶^	83.07
Malodor salivary organisms	Control	6.72 ± 0.61	6.09 ± 0.66 ^¶^	76.50
	Gingivitis	6.82 ± 0.49	6.17 ± 0.54 ^¶^	77.92
	Halitosis	6.86 ± 0.55	6.13 ± 0.46 ^¶^	81.34

^ǂ^ No significant differences between treatment groups for evaluated organism (*p* > 0.05). ^¶^ Significantly different from corresponding baseline by paired *t*-test for evaluated organism (*p* < 0.05).

**Table 7:** Summary of tongue surface organisms (Log CFU/mL) for subjects who completed the clinical study.

Bacteria Evaluated	Treatment Groups Based on Clinical Status	Baseline Visit ^ǂ^	2-Week Post-Treatment Visit ^¶^	Percent Reduction from Corresponding Baseline
Anaerobic tongue organisms	Control	7.08 ± 0.73	6.43 ± 0.70 ^¶^	77.72
	Gingivitis	7.54 ± 0.53	6.76 ± 0.82 ^¶^	83.44
	Halitosis	7.63 ± 0.39	6.81 ± 0.71 ^¶^	85.01
Malodor tongue organisms	Control	6.64 ± 0.46	6.12 ± 0.55 ^¶^	69.45
	Gingivitis	6.75 ± 0.49	6.28 ± 0.55 ^¶^	66.73
	Halitosis	6.75 ± 0.50	6.07 ± 0.64 ^¶^	79.39

^ǂ^ No significant differences between treatment groups for evaluated organism (*p* > 0.05). ^¶^ Significantly different from corresponding baseline by paired *t*-test for evaluated organism (*p* < 0.05).

## Data Availability

Study subjects did not consent to public date sharing, so supporting data is not available.
